# AI-Predicted Model-Guided Rebuilding of the Experimental Structure of Mouse δ-Aminolevulinic Acid Dehydratase

**DOI:** 10.3390/ijms27156553

**Published:** 2026-07-23

**Authors:** Ki Hyun Nam

**Affiliations:** College of General Education, Kookmin University, Seoul 02707, Republic of Korea; structure@kookmin.ac.kr

**Keywords:** artifact intelligent, experimental structure, rebuilding, δ-aminolevulinic, alphafold

## Abstract

Experimental macromolecular structures are foundational for elucidating molecular mechanisms and guiding drug design and protein engineering. However, inaccurate structural models are occasionally deposited in the Protein Data Bank (PDB), potentially confounding structural analyses and misleading subsequent studies. Although the integration of artificial intelligence (AI)-predicted models to improve experimentally determined structures has been proposed, the impact of AI-guided rebuilding on functional interpretation remains largely uncharacterized. Here, AI-predicted models of δ-aminolevulinic acid dehydratase (ALAD) were analyzed, and a misinterpreted region in its original crystal structure was rebuilt using an AlphaFold3 (AF3) model as a template. The incorrectly modeled region between Cys122 and Leu142 was corrected utilizing the AF3 main-chain conformation. This rebuilding decreased the R_free_ value and improved structural geometry compared to the experimental structure. Notably, the initially deposited ALAD structure exhibited an inactive conformation characterized by a disrupted substrate-binding A-site. In contrast, the AI-rebuilt ALAD model restored a biologically relevant active-site conformation, featuring a coordinated zinc-binding pocket, mediated by Cys122, Cys124, and Cys132, and an intact substrate-binding configuration at the A-site. These results demonstrate the feasibility of AI-predicted model-guided rebuilding in the present ALAD case and may provide a basis for future studies evaluating its applicability to other protein systems.

## 1. Introduction

Experimentally determined macromolecular structures, freely accessible through the Protein Data Bank (PDB) [1,2], provide valuable insights into molecular properties such as ligand binding, protein–protein interactions, and catalytic reactions [3,4]. Such structural information is important for understanding biological functions [5,6] and drives various downstream applications, such as molecular dynamics simulations [7,8,9], structure-based drug design [10,11,12], and rational protein engineering [13,14,15,16]. Therefore, the accuracy of these deposited models is critical for ensuring the reliability and success of subsequent studies.

Despite their broad utility, inaccurate model building and suboptimal refinement statistics are frequently observed in among PDB depositions [16,17,18,19,20]. These inaccuracies are often associated with low-resolution data, radiation damage, or high molecular flexibility, which yield ambiguous or weak electron density maps [21,22,23,24]. Furthermore, differences in researchers’ model-building expertise can compromise the overall quality of the final structure [25]. Because relying on inaccurate structural models may result in misinterpretation of molecular function and research design, validation and systemic correction of experimentally determined structures are necessary.

Recently, AI-based structure prediction tools, such as AlphaFold2 (AF2) [26], AlphaFold3 (AF3) [27], ESMFold (ESM) [28], and RoseTTAFold (RF) [29], have achieved remarkable accuracy and see widespread application across structural biology and biotechnology [30]. In X-ray crystallography, AI-predicted structures routinely serve as search models for molecular replacement to overcome the phase problem [30,31], while in cryo-electron microscopy (cryo-EM), they provide initial templates to assist model building [32,33]. Meanwhile, recent AI-assisted approaches, including ModelAngelo and the DEMO-EM series, have facilitated automated model building and refinement in cryo-EM studies [34,35,36,37,38]. Beyond experimental structure determination, AI-predicted models facilitate basic protein function investigations [39,40,41] and are increasingly employed in docking simulations during early-stage inhibitor design [42,43]. Together, these AI-based models have significantly advanced broad areas of biological and biomedical research.

In particular, the integration of AI-predicted models to refine and correct existing experimentally determined structures has been previously proposed [25]. For example, a prior study utilized AlphaFold2 models to tentatively rebuild missing residues in the lumenal domain of calnexin (PDB ID: 1JHN) [44] and correct backbone tracings in δ-aminolevulinic acid dehydratase (ALAD; PDB ID: 2Z1B, unpublished) [25]. However, these studies primarily focused on backbone-level improvements without comprehensively analyzing how such corrections affect overall model accuracy or subsequent biological functional interpretation.

To investigate the utility of AI-predicted model-guided structural refinement, AI-predicted ALAD models generated by ESM, AF2, AF3, and RF were analyzed. The crystal structure of *Mus musculus* ALAD was rebuilt using the AF3 model as a template, and the refinement statis-tics were compared. By comparing the rebuilt regions and active sites against the original experimental model, biological relevance of the corrected ALAD conformation was assessed. This study demonstrates the feasibility of AI-predicted model-guided rebuilding for correcting modeling errors in the present ALAD structure and elucidates the biologically functional state of ALAD.

## 2. Results

### 2.1. Revisiting the ALAD Structure

**Figure 1:**
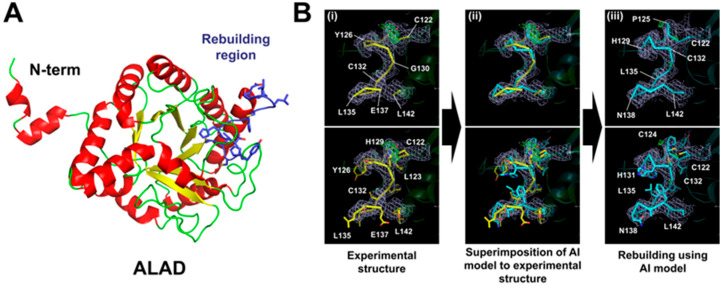
Revisiting the experimental ALAD structure and AI-guided rebuilding. (**A**) Cartoon representation of the *Mus musculus* ALAD structure (PDB ID: 2Z1B, chain B). The rebuilt region is indicated in blue sticks and cartoon format. (**B**) Procedure for improving the experimental structure using the AI-predicted model. (**i**) Experimental ALAD structure (chain B) superimposed on the 2Fo−Fc (gray mesh, 0.9σ) and Fo−Fc (green mesh, +3σ) electron density maps. The region spanning Glu137 and Leu142 was absent in the original PDB coordinates. (**ii**) Superimposition of the AI-predicted ALAD model (cyan) onto the experimental ALAD structure (yellow). (**iii**) Structure refinement using the AI-predicted model and the experimental electron density map. Panel B is reproduced from a previous report [25].

Two crystal structures of ALAD from *Mus musculus* are currently deposited in the PDB: the native form (PDB ID: 2Z1B, resolution: 3.3 Å) and the selenomethionine-derivatized form (2Z0I, 3.1 Å), which contain four and two molecules in the asymmetric unit, respectively. The overall structure of ALAD exhibits a TIM-barrel fold with an additional N-terminal α-helical region (Figure 1A). Several missing residues localize to loop regions in the native form of ALAD (Val85–Ala94, Tyr126–Phe141, and Ser214–Cys223 in chain A; Asn138–Phe141 in chain B; Val85–Ala94, Cys124–Phe141, and Ser214–Cys223 in chain C; and Val85–Asp95, Cys124–Phe141, and Ser214–Cys223 in chain D) and selenomethionine-derivatized ALAD (Val85–Asp96, Cys124–Leu141, and Ser214–Cys223 in chain A; and Ser83–Asp95, Cys124–Leu142, and Arg209–Cys223 in chain B) (Supplementary Figure S1). Inspection of the electron density maps of ALAD confirmed that these missing-residue regions generally lack an interpretable electron density map.

However, chain B of native ALAD presents a relatively higher electron density map quality than the other chains in both native and selenomethionine-derivatized ALAD structures. Specifically, native chain B contains only a single short stretch of four missing residue between Glu137 and Leu142. Although the electron density map in this region is relatively weak, the surrounding main chain remained traceable, allowing reconstruction of both the missing segment and the incorrectly modeled region. Accordingly, reliable model building of native chain B can provide a reliable full-length structure of *Mus musculus* ALAD. Throughout this study, structural analyses focus primarily on the chain B molecule, except during the structure refinement of all molecules in the asymmetric unit; therefore, all subsequent ALAD structural descriptions herein refer to native chain B unless otherwise stated.

Notably, the Cα–Cα distance between Glu137 and Leu142, excluding the missing residues, is approximately 5.0 Å. This distance is insufficient to accommodate the four missing residues (Figure 1B), suggesting a misinterpretation of the main-chain backbone [25]. A previous study noted that an AF2-predicted model fits well into the existing electron density map of ALAD, supporting reliable backbone tracing of the missing-residue region [25]. This previous study demonstrated that the 24-residue region from Cys122 to Cys145 in ALAD deposited in the PDB may have been modeled incorrectly [25]. Although that report highlighted the potential utility of AI-predicted models for improving experimental structures, it did not comprehensively evaluate the reliability of the rebuilt structure. In particular, it remains unclear whether such AI-guided structural corrections yield a biologically relevant, functional conformation.

### 2.2. Preparation of the AI-Predicted Model

A previous study utilized an AF2-predicted model to re-trace the main-chain of ALAD [25]. To date, various other AI-based structure prediction tools, such as AF3, ESM, and RF, have been developed. To identify the most suitable model for rebuilding the ALAD structure and investigate structural differences among AI-predicted models, ALAD structures generated using AF3, ESM, and RF were evaluated for their structural quality and compatibility with the experimental ALAD structure.

**Figure 2:**
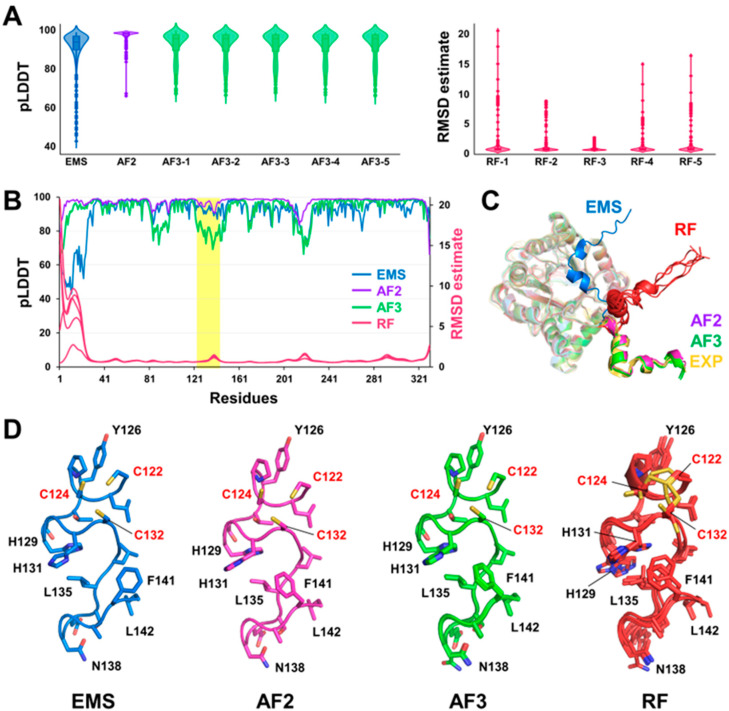
Evaluation of the AI-predicted ALAD models generated by ESM, AF2, AF3, and RF. (**A**) Violin plots showing the pLDDT confidence scores for models generated using ESM, AF2, and AF3 (AF3-1 to AF3-5) and the predicted Ångström error estimates for RF (RF-1 to RF-5). (**B**) Residue-wise plots of pLDDT values for ESM, AF2, and AF3 and predicted Ångström error estimates for RF. The rebuilding region is highlighted in yellow. ESM-generated pLDDT scores were normalized to a 0–100 percentage scale. (**C**) Superimposition of the AI-predicted ALAD structures, generated using ESM (blue), AF2 (purple), AF3 (green), and RF (red), onto the original crystal structure of ALAD (yellow; PDB ID: 2Z1B, chain B). (**D**) Close-up view of the rebuilt region (Cys122–Leu142) across the AI-predicted ALAD structures by ESM, AF2, AF3, and RF. The AF3 and RF panels show superimpositions of the five predicted model structures. The three cysteine residues (Cys122, Cys124, and Cys132) among the AI-predicted models are indicated in red font.

ESM, AF2, AF3, and RF generated 1, 1, 5, and 5 ALAD models, respectively. The predicted local distance difference test (pLDDT) scores of the ESM-ALAD model ranged from 0.43 to 0.99, with an average value of 0.93 (Figure 2A). The pLDDT values for the AF2-ALAD model ranged from 67.56 to 98.94, with an average value of 98.07 (Figure 2A). The pLDDT values of the five AF3-ALAD models ranged from 66.81 to 98.78, with average values of 96.21–96.22 (Figure 2A). Superimposition of the five AF3-ALAD models showed very high structural similarity, with RMSDs ranging from 0.049 to 0.105 Å (Figure 2A). The predicted Ångström error estimates of the five RF-ALAD models ranged from 0.49 to 20.71, with average values of 0.78–0.84 (Figure 2A). Superimposition of the five RF-ALAD models showed RMSDs ranging from 0.159 to 0.254 Å (Figure 2A), indicating greater structural variation among the RF models than the AF3 models. Although all models exhibited high confidence scores based on pLDDT or predicted Ångström error estimates, the confidence regions varied depending on the AI prediction tool and individual model (Figure 2B). Regions with relatively low pLDDT scores in the ESM, AF2, and AF3 models or high predicted Ångström error estimates in the RF models were not completely consistent, indicating structural differences among the AI-predicted models. Overall, the AF2 model exhibited higher confidence scores across most regions, followed closely by the ESM model (Figure 2B). The five AF3-predicted models displayed highly similar pLDDT profiles throughout the amino acid sequence; however, the N-terminal predicted Ångström error estimates of the RF-ALAD models varied substantially among the individual models (Figure 2B). Notably, in all AI-predicted models, the missing-residue region between Cys122 and Leu142 consistently exhibited one of the lowest confidence score regions compared with the remaining regions of the structure.

Superimposition of the ESM-, AF2-, AF3-, and RF-predicted ALAD structures onto the experimental ALAD structure (PDB ID: 2Z1B) yielded RMSDs of 0.649 Å, 0.505 Å, 0.442–0.457 Å, and 0.821–0.851 Å, respectively. The N-terminal regions of the AF2 and AF3 models aligned well with the experimental ALAD structures, whereas the N-terminal conformations of the ESM- and RF-predicted ALAD models exhibited noticeable N-terminal differences (Figure 2C). Despite these N-terminal variations, the main-chain positions of the rebuilding region between Cys122 and Leu142 were nearly identical across all AI-predicted model structures (Figure 2D). Furthermore, the side-chain conformations within the rebuilding region were also highly conserved among the ESM, AF2, and AF3 models, with minor differences in the side-chain conformations of Asn138 and Phe141. In contrast, the RF models displayed substantial differences in the side-chain positions and conformations compared to those of the other AI-predicted structures (Figure 2D). Within the rebuilding region of ALAD, all models oriented the side chains of Cys122, Cys124, and Cys132 in the same direction. In the ESM, AF2, and AF3 models, the respective sulfhydryl groups remained separated by distances greater than 2.8 Å (Figure 2D). In contrast, the RF models predicted disulfide bond formation between Cys122, Cys124, and Cys132 with distances ranging from 2.0 to 2.1 Å, whereas the remaining non-disulfide-bonded cysteine residues were oriented away from the center of the cysteine cluster (Figure 2D and Supplementary Figure S2). These results indicate that detailed side-chain positions and conformations differ among AI-predicted models and prediction tools.

### 2.3. Rebuilding the ALAD Structure Using the AF3 Structure

**Figure 3:**
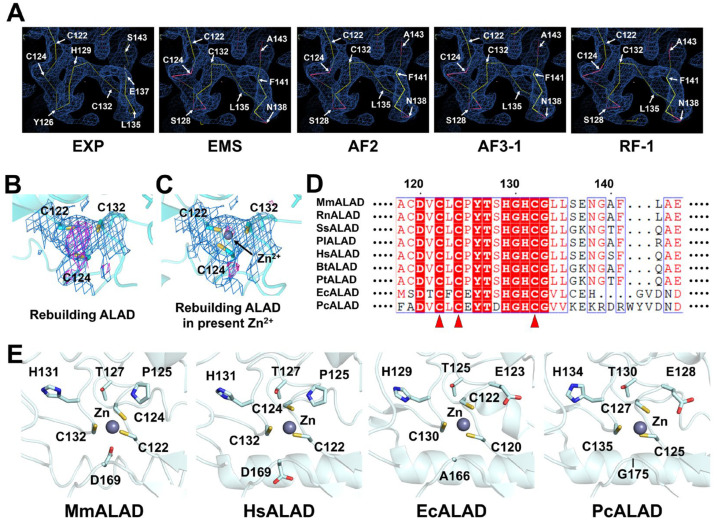
Rebuilding the ALAD zinc-binding site and homologous structural comparisons. (**A**) AI-predicted ALAD models superimposed onto the 2Fo−Fc (blue mesh, 0.9σ) and Fo−Fc (green mesh, 3σ; red mesh, −3σ) electron density maps of the experimental ALAD structure (PDB ID: 2Z1B). (**B**) Rebuilt ALAD structure fitted to the 2Fo−Fc (blue mesh, 0.8σ) and Fo−Fc (magenta mesh, 5σ) electron density maps of the experimental ALAD structure. (**C**) Refinement of the rebuilt ALAD model in the presence of zinc revealed no significant positive Fo−Fc electron density. (**D**) Partial sequence alignment of the rebuilt region across ALAD orthologs: *Mus musculus* (MmALAD, UniProt code: P10518), *Rattus norvegicus* (RnALAD, P06214), *Suricata suricatta* (SsALAD, A0A673UVC7), *Panthera leo* (PlALAD, A0A8C8XR68), *Homo sapiens* (HsALAD, P13716), *Bos taurus* (BtALAD, Q58DK5), *Panthera tigris altaica* (PtALAD, A0A8C9M8G4), *Pyrobaculum calidifontis* JCM 11548 (PcALAD, A3MWV9), and *Escherichia coli* (EcALAD, P0ACB2). The three cysteine residues essential for zinc coordination are indicated by red triangles. (**E**) Structural comparison of the rebuilt MmALAD against the zinc-binding sites of HsALAD (PDB code: 5HNR), EcALAD (PDB code: 5MHB), and PcALAD (PDB code: 5LZL).

Superimposition of the AF2, AF3, ESM, and RF models onto the experimental structure revealed that the AI-predicted model structures were well fitted within the electron density map of the experimental ALAD structure (Figure 3A and Supplementary Figure S3). To correct the mismodeled region between Cys122 and Leu144 in chain B of ALAD (named EXP-ALAD), the AF3-predicted ALAD structure (AF3-ALAD) was used as the primary template (Figure 3B), as its side chains in the rebuilt region exhibited superior fit to the electron density map compared with those of the other AI model structures. The overall rebuilding process was similar to that of the previously established methods (Figure 1B). Briefly, an AF3-ALAD structure was superimposed onto EXP-ALAD, and then the experimental incorrect regions (Cys122-Ley142) were replaced by AF3-ALAD. The rebuilding region of AF3-ALAD, including three cysteine residues (Cys122, Cys124, and Cys132), was well fitted into the 2Fo-Fc electron density map (Figure 3B). After rigid-body refinement, the ALAD model was manually rebuilt and subjected to further structural refinement to generate the final rebuilt ALAD (RE-ALAD) structure. Although specific side-chain positions remained ambiguous owing to poor electron density or intrinsic flexibility, the overall modeled structure, particularly the main chain in the rebuilt region from Cys122 to Leu142, fit well into the electron density map (Supplementary Figures S4 and S5).

During the refinement of RE-ALAD, positive Fo–Fc electron density peaks (>5σ) were observed adjacent to the sulfhydryl groups of Cys122, Cys124, and Cys132 (Figure 3B). The distances between the sulfhydryl groups of Cys122–Cys124, Cys122–Cys132, and Cys124–Cys132 were longer than 2.8 Å in the rebuilt ALAD structure. These results suggest the possible coordination of a heavy metal ion.

To determine evolutionary conservation, the sequence alignment of *Mus musculus* ALAD was performed with homologous ALAD sequences from *Rattus norvegicus* (RnALAD; sequence identity: 97.3%, residue overlap: 330 a.a.), *Suricata suricatta* (SsALAD; 90.6%, 330 a.a.), *Panthera leo* (PlALAD; 89.4%, 330 a.a.), *Homo sapiens* (HsALAD; 89.1%, 330 a.a.), *Bos taurus* (BtALAD; 89.7%, 329 a.a.), *Panthera tigris altaica* (PtALAD; 86.8%, 334 a.a.), *Escherichia coli* (EcALAD; 44.0%, 300 a.a.), and *Pyrobaculum calidifontis* JCM 11548 (PcALAD; 44.1%, 313 a.a.). This alignment confirmed that Cys122, Cys124, and Cys132 in *Mus musculus* ALAD are highly conserved among ALAD homologs (Figure 3C). Among them, HsALAD is identified as a zinc-dependent enzyme [16], in which the zinc ion is coordinated by Cys132, Cys134, and Cys142 (PDB ID: 5HNR) [45]; these residues are sequentially and positionally conserved with ALAD (Figure 3D). Similar zinc-coordinating cysteine triads are documented in PcALAD (Cys125, Cys127, and Cys135; PDB ID: 5LZL) [45] and EcALAD (Cys132, Cys134, and Cys142; PDB ID: 5MHB) [45]. These cysteine residues are sequentially and structurally conserved compared with those in ALAD (Figure 3D). These results indicate that the positive electron density map observed above the three cysteine residues in the rebuilt ALAD structure corresponds to a zinc ion.

To verify zinc ion binding to the three cysteine residues in ALAD, structural refinement of ALAD was performed in the presence of a zinc. The results showed that the significant positive Fo–Fc electron density map disappeared, and no negative Fo–Fc electron density map was observed around the three cysteine residues in ALAD (Figure 3B), indicating zinc binding to the experimental ALAD structure.

To validate the accuracy of the AF3-guided RE-ALAD model, refinement statistics were compared between EXP-ALAD and RE-ALAD. Because modern refinement programs yield better refinement statistics than those obtained using older refinement procedures, structure refinement was performed for RE-ALAD and EXP-ALAD under identical refinement parameters (Table 1). The de-posited EXP-ALAD structure showed R_work_ and R_free_ values of 0.278 and 0.353, respectively, whereas re-refinement of EXP-ALAD using a modern refinement program improved these values to 0.2275 and 0.3128, respectively. Meanwhile, RE-ALAD exhibited R_work_ and R_free_ values of 0.2270 and 0.3098, respectively. This further decrease in R values, compared with those of the re-refined EXP-ALAD structure, suggests that RE-ALAD was more accurate than EXP-ALAD.

**Figure 4:**
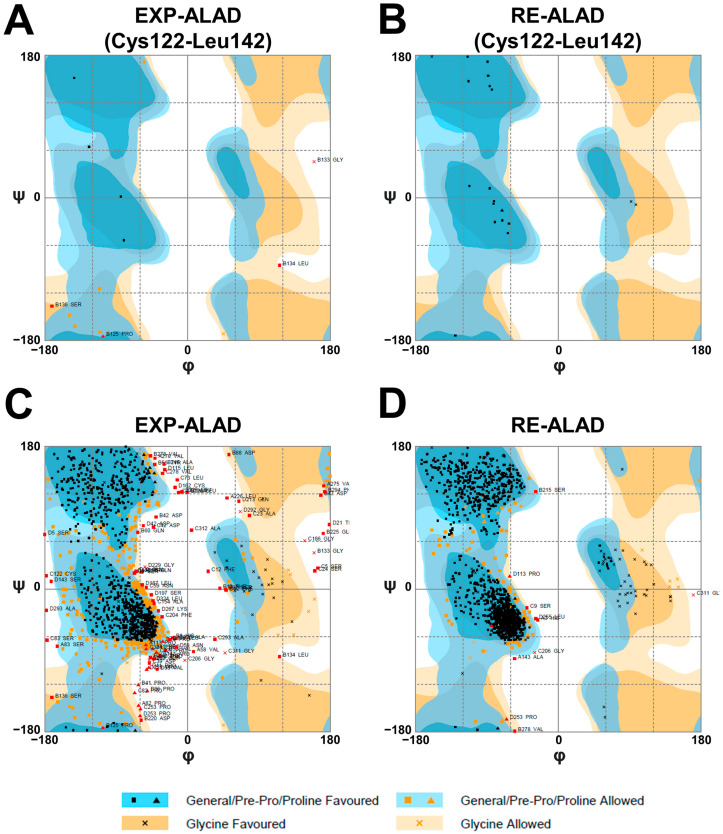
Ramachandran plots of EXP-ALAD and RE-ALAD. Geometry of the specific rebuilt region (Cys122–Leu144) in (**A**) EXP-ALAD (PDB ID: 2Z1B) and (**B**) RE-ALAD. Overall stereochemical geometry of the four ALAD molecules in the asymmetric unit for (**C**) EXP-ALAD and (**D**) RE-ALAD.

Next, the geometry of the rebuilt region was evaluated using Ramachandran plot analysis. In EXP-ALAD, the specific target region displayed poor geometry, with only 28.6% (4 of 14 residues) and 42.9% (6 of 14) of residues localizing to favored and allowed regions, respectively. Furthermore, 28.6% (4 of 14) of the residues, including Pro125, Gly133, Leu134, and Ser136, were classified as outliers (Figure 4A), indicating poor backbone geometry in the rebuilt region. In contrast, all 19 residues in RE-ALAD were located in favored regions (Figure 4B), indicating that RE-ALAD exhibits substantially improved backbone geometry in the rebuilt region.

Meanwhile, the initial RE-ALAD structure still exhibited a relatively high R_free_ value, likely because only a subset of the four molecules in the asymmetric unit had been rebuilt. Accordingly, all four ALAD molecules in the asymmetric unit were subsequently rebuilt using the AF3-ALAD structure as a template. Following the superimposition, initial rigid-body refinement, and further manual refinement, residues lacking clear main-chain electron density in the 2Fo−Fc electron density map at 0.85σ were removed from the model structure. Refinement of the rebuilt four-molecule ALAD structure significantly lowered R_work_ and R_free_ values to 0.2122 and 0.2749, respectively (Table 1). Ramachandran plot analysis confirmed that 90.10%, 8.74%, and 1.16% of total residues were located in favored, allowed, and disallowed regions, respectively (Figure 4). The average RSCC values of Cys122–Leu142 for EXP-ALAD, re-refined EXP-ALAD, and RE-ALAD were 0.731, 0.797, and 0.901, respectively, indicating an improved local map-to-model fit quality after AI-guided rebuilding. Together, these results indicate that AI-guided rebuilding successfully resolved the widespread tracing errors and poor geometry present in the original EXP-ALAD deposition.

### 2.4. Structural Comparison of the Rebuilding Regions of RE-ALAD and EXP-ALAD

**Figure 5:**
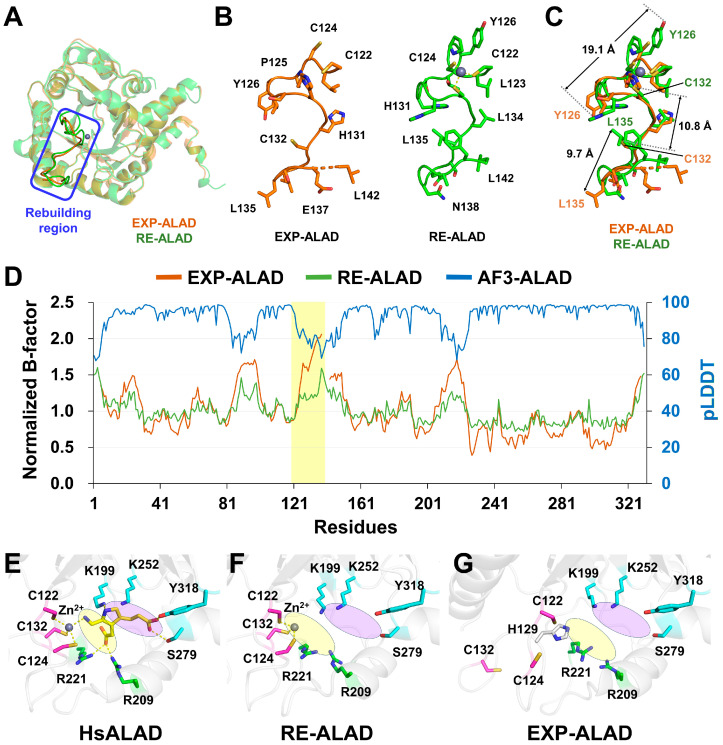
Structural comparison of EXP-ALAD and RE-ALAD. (**A**) Superimposition of the EXP-ALAD (orange; PDB ID: 2Z1B) and RE-ALAD (green) structures. (**B**) Close-up view comparing the rebuilt region between EXP-ALAD and RE-ALAD. (**C**) Superimposition of the rebuilding regions from EXP-ALAD (orange) and RE-ALAD (green). (**D**) Normalized B-factor plot of EXP-ALAD (orange; chain B) and RE-ALAD (green), overlaid with the pLDDT values of AF3-1 model (blue). (**E**) Crystal structure of the human ALAD substrate-binding site in complex with porphobilinogen (PDB ID: 1E51). Substrate-binding sites of (**F**) RE-ALAD and (**G**) EXP-ALAD. The substrate δ-ALA-binding A- and P-sites are colored yellow and magenta, respectively. The three cysteine residues involved in zinc binding are shown as magenta sticks. The δ-ALA-binding residues at the A and P sites are displayed as green and cyan sticks, respectively.

To evaluate how AI-guided structural rebuilding impacts differences functional interpretation, the EXP-ALAD and RE-ALAD structures were compared. Superimposition of the EXP-ALAD and RE-ALAD structures revealed high structural similarity, with an RMSD value of 0.365 Å (Figure 5A), indicating that the overall fold remains highly conserved. However, subtle conformational and positional differences were observed in the N-terminal and loop regions. In particular, the 21-residue segment within the rebuilt region between Cys132 and Leu142 exhibited significant structural reorganization (Figure 5A). In EXP-ALAD, the sulfur atom of Cys122 involved in zinc coordination was located near the zinc-binding site at a distance of 2.92 Å, whereas the sulfur atoms of Cys124 and Cys132, which are also involved in zinc coordination, were severely displaced by approximately 8.7 and 12.9 Å, respectively. This could potentially lead to the incorrect interpretation that zinc is not natively coordinated by the three cysteine residues (Figure 5B). In contrast, RE-ALAD successfully restored the conserved zinc-binding configuration among Cys122, Cys124, and Cys132 (Figure 5B). The distances between the corresponding OH atom of Tyr126, SG atom of Cys132, and CG atom of Leu135 in EXP-ALAD and RE-ALAD were 19.1, 10.8, and 9.7 Å, respectively (Figure 5C).

Normalized B-factor analysis showed that the mismodeled region of EXP-ALAD exhibited the highest flexibility in the entire polypeptide chain, reaching a peak normalized B-factor value of 2.06 (Figure 5D). Although the rebuilt region of RE-ALAD remained relatively flexible, its peak normalized B-factor value decreased substantially to 1.59, a value comparable to those of the N- and C-terminal regions (Figure 5D). Meanwhile, regions with relatively high B-factor values in ALAD corresponded to regions showing relatively low pLDDT confidence scores in the AF3 model (Figure 5D).

Next, the effects of structural differences in the rebuilt region between EXP-ALAD and RE-ALAD on functional interpretation were investigated. ALAD catalyzes the second step of the heme biosynthesis pathway, mediating the asymmetric condensation of two molecules of δ-aminolevulinic acid (δ-ALA) into porphobilinogen [46]. During the enzyme reaction, two δ-ALA molecules bind to two distinct substrate-binding sites, referred to as the A-site and P-site, corresponding to the acetyl and propionyl groups of the final porphobilinogen product, respectively [47]. Binding of δ-ALA to the A-site specifically induces closure of the active-site lid and necessitates coordination with the catalytic zinc ion [47].

In the homologous crystal structure of human ALAD complexed with porphobilinogen (PDB ID: 1E51), the P-site substrate is coordinated by Ser279, Tyr318, Lys199, and Lys252. In contrast, the A-site substrate is coordinated by Arg209, Arg221, and the zinc ion bound by the three cysteine residues Cys122, Cys124, and Cys132 (Figure 5E). The main-chain positions of these substrate-binding residues and the corresponding zinc-binding site in RE-ALAD were highly similar to those in human ALAD (Figure 5F). This indicates that RE-ALAD adopts a biologically relevant apo-state conformation capable of accommodating δ-ALA binding at both the A- and P-sites. In contrast, the mismodeled backbone in EXP-ALAD completely disrupts the zinc-binding site, rendering the A-site structurally incompatible for proper δ-ALA binding, although the positions of residues interacting with the P-site substrate were largely conserved (Figure 5G). Therefore, RE-ALAD represents a biologically relevant apo-state structure, whereas EXP-ALAD exhibits a structurally unreliable active-site conformation.

## 3. Discussion

The deposition of accurate structural coordinates in the PDB is important for subsequent research, such as functional analysis, drug design, and protein engineering. To avoid misinterpretation and misleading conclusions in subsequent research, it is essential to identify inaccurate and correct inaccurate structural models. In a previous study, it was proposed that the incorrectly modeled structure of ALAD could be corrected using an AI-predicted model structure [25]. However, the effects of these corrections on data quality and their structural significance were not investigated. Therefore, it remains unclear to what extent AI-based model refinement contributes to improving structure quality and providing meaningful structural insights.

This study extends the previous concept of AI-guided structural refinement using the experimental structure of *Mus musculus* ALAD. The coordinates of originally deposited EXP-ALAD fit poorly within the experimental electron density map, indicating severe mismodeling in the active site. In contrast, the main chain of ESM-, AF2-, AF3-, and RF-predicted ALAD models fits well within the electron density map of the experimental ALAD structure, thereby providing a reliable template for the active-site region of the ALAD model. In particular, the AF2, AF3, and ESM models predicted side-chain conformations in which the three cysteine residues in the active site properly coordinated a zinc ion. In contrast, the RF models erroneously predicted disulfide bond formation in some zinc-coordinating cysteine residues, indicating that AI model accuracy differed depending on the AI prediction tool. Despite these localized inaccuracies in the RF models, such issues could be corrected during manual remodeling. Accordingly, the AI-predicted ALAD models obtained in this study exhibited high structural reliability and good agreement with the experimental electron density maps.

RE-ALAD using AI-predicted models significantly reduced the R_work_ and R_free_ values compared with the original EXP-ALAD structure, indicating that the AI-predicted models served as effective rebuilding templates for ALAD. In particular, while the EXP-ALAD structure exhibited poor Ramachandran plot geometry, RE-ALAD structure using AF3 was close to the ideal geometry. Meanwhile, although the statistics of the original EXP-ALAD structure were also improved by refinement using modern refinement programs, the AI-guided RE-ALAD structure achieved superior overall accuracy.

Interestingly, although these AI-based prediction programs were trained on the existing PDB datasets, they did not reproduce the incorrectly modeled ALAD structure deposited in the database. Instead, they accurately predicted the biologically functional zinc-binding conformation of ALAD. This observation suggests that AI prediction tools do not simply replicate existing PDB structures; rather, they infer structurally and functionally plausible conformations based on broadly learned structural features, consistent with previous reports [26,29].

The structural differences between EXP-ALAD and the AI-guided rebuilt ALAD structure led to different interpretations of the ALAD function. The mismodeling of the active site in the EXP-ALAD structure displaced the zinc-coordinating cysteine residues. Consequently, the EXP-ALAD structure presents an inactive active-site conformation that could easily lead to the misleading interpretation that the zinc-binding site of ALAD adopts multiple conformations in the absence of metal ions. In contrast, the RE-ALAD model clearly showed that the three cysteine residues coordinate with a zinc ion. This rebuilt structure exhibited high structural similarity to the previously reported functional state of ALAD structures from other organisms, confirming that this zinc-binding configuration of ALAD, along with its capacity for substrate binding, is highly conserved. Therefore, the RE-ALAD structure provides a more reliable structural model for future biological studies of *Mus musculus* ALAD.

Meanwhile, the AI-predicted model structures in this study provided highly accurate structures that align well with the electron density map of the experimental ALAD; however, the AI-predicted model does not always generate accurate structures [48,49,50]. In general, although AI-predicted protein structures exhibit highly accurate overall folding, localized side-chain conformations can exhibit variations [48,51]. For example, the overall folding of HviGH11 xylanase, which had not previously been experimentally determined, showed high similarity to AI-predicted structures [52]. However, the AF2, AF3, and ESM models exhibited subtle deviations and minor conformational changes in the side chains within the substrate-binding site. Moreover, the RF model produced side-chain conformations inconsistent with the experimentally determined structure and biological function [52]. These findings indicate that the similarity between AI-predicted and experimental structures may vary depending on both the specific AI tool used and the target protein.

Moreover, current AI algorithms struggle to accurately predict post-translational modifications or environmentally induced structural changes [53]. For example, current AI prediction tools fail to predict chromophore formation in fluorescent proteins generated through post-translational modification of tripeptides and do not accurately reproduce light- or pH-dependent conformational changes in chromophores [54,55]. Because AI-predicted structures do not explicitly consider environmental factors such as water molecules, buffer conditions, or pH, they cannot completely replace experimentally determined structures [56]. In addition, AI-predicted models are inherently static representations and may not fully capture the conformational flexibility associated with protein function [57,58]. Accordingly, the applicability of AI-guided structural rebuilding should be carefully evaluated for proteins that undergo conformational transitions or adopt different conformations. However, these structural inaccuracies can often be resolved during manual model-building procedures.

Meanwhile, in the case of ALAD, homologous experimental structures from other organisms contain a highly conserved zinc-binding site and could also have been used as rebuilding templates for ambiguous regions, indicating that an AI-predicted model was not strictly necessary in this case. However, AI-based rebuilding may be particularly valuable for proteins lacking suitable experimentally determined homologous structures, where reliable template structures are unavailable.

Further studies using a broader range of protein systems will be required to determine whether this approach can be generally applied to experimentally determined structures deposited in the PDB, particularly those containing modeling errors or regions with ambiguous electron density. Such studies will provide further insight into the applicability and limitations of AI-predicted models as rebuilding templates and may contribute to improving the efficiency and accuracy of structure rebuilding. Whether this AI-predicted model-guided rebuilding approach can be broadly generalized remains to be established through analyses of additional protein systems encompassing diverse folds, resolutions, and structural challenges. Ultimately, more accurate structural models will facilitate the interpretation of protein structure and function and provide a more reliable foundation for subsequent structural and functional studies.

## 4. Materials and Methods

### 4.1. Modeling of the ALAD Suing AI Prediction Tools

The amino acid sequence of *Mus musculus* ALAD (UniProt accession code: P10518) was retrieved from the UniProtKB database [59]. Structural models of ALAD were generated using the AlphaFold3 [27], ESMFold [28], and RoseTTAFold [29] servers using the amino acid sequence of ALAD as the input, with default parameters. AlphaFold3 and RoseTTAFold generated five predicted models, whereas ESMFold provided a single predicted model. The AlphaFold2-predicted ALAD model was obtained directly from the AlphaFold Protein Structure Database (https://alphafold.ebi.ac.uk/), which provides a single predicted structural model. The confidence of the model structures of AlaphFold2, AlphaFold3 and ESMfold was evaluated by pLDDT values and confidence of the model structures of RoseTTAFold was evaluated by estimated RMSD values.

### 4.2. Rebuilding the ALAD Structure

Structure factors and atomic coordinates of ALAD (PDB IDs: 2Z1B and 2Z10) were obtained from the PDB [2]. The AF3-predicted model structure was superimposed onto the experimental ALAD structure using the COOT program [60]. The positions of the Cα atoms were visually inspected against the experimental 2Fo-Fc and Fo-Fc electron density maps. The coordinates spanning Cys122–Leu142 in chain B of ALAD (PDB ID: 2Z1B) were replaced with those of the corresponding residues from the AF3 model. Following the initial rigid-body refinement of this rebuilt region, side-chain conformations were manually adjusted to fit the density. Real-space refinement of the replaced region (Cys122–Leu142) was subsequently conducted using COOT (v. 0.9.8.96) [60]. Final structural refinement was performed using phenix.refine in the PHENIX (v. 1.20.1-4487) software suite [61]. For rebuilding the four ALAD molecules in the asymmetric unit, four identical AF3-predicted models were superimposed onto the corresponding experimental ALAD molecules, and manual model rebuilding was performed based on the 2Fo−Fc and Fo−Fc electron density map. During refinement, atomic coordinates were built only where the 2Fo−Fc electron density map was clearly observed at the 0.8σ contour level. Regions with ambiguous or poorly defined electron density were not built. The geometry of the experimental and rebuilt structures was validated using MolProbity [62].

### 4.3. Bioinformatics

Homologous ALAD sequences were identified using BLAST [63]. Multiple sequence alignments of ALAD and homologous proteins were generated using Clustal Omega [64]. Structure-based sequence alignments were visualized using ESPript 3.0 [65]. Structural figures were rendered using PyMOL (v. 2.5.10) (https://pymol.org). Ramachandran plots were generated using RAMPAGE [66] as implemented in the CCP4 Cloud suite [67].

## 5. Conclusions

This study demonstrates that biologically relevant ALAD structures can be accurately reconstructed from misbuilt experimental structures using AI-predicted models as rebuilding templates. This finding provides a proof of concept for AI-predicted model-guided rebuilding in the present ALAD case, while further studies will be required to determine whether this approach is applicable to other protein systems.

## Figures and Tables

**Table 1:** Refinement statistics of re-refined ALAD and rebuilding ALAD using the AF3 model.

	EXP-ALAD	Re-Refined EXP-ALAD	RE-ALAD (C122–L142, Chain B)	RE-ALAD (Chain A–D)
Resolution (Å)	43.40–3.30	43.39–3.30	43.39–3.30	43.39–3.30
Refinement program (version)	CNS (1.1)	PHENIX (1.20.1_4487)	PHENIX (1.20.1_4487)	PHENIX (1.20.1_4487)
R_work_	0.278	0.2275	0.2270	0.2122
R_free_	0.353	0.3128	0.3098	0.2749
B-factor (Å^2^)				
Protein	71.09	87.93	88.78	93.79
Waters	25.20	76.64	76.60	
R.m.s deviations				
Bond lengths (Å)	0.006	0.011	0.011	0.011
Bond angles (°)	1.200	1.489	1.484	1.381
Ramachandran plot (%)				
Favored	64.7	72.69	73.34	90.10
Allowed	24.6	19.09	18.91	8.74
Disallowed	10.7	8.22	7.75	1.16
Real-Space Correlation Coefficient (Chain B, Cys122–Leu142)	0.731	0.797	0.901	0.901

## Data Availability

The model structures of ALAD generated by ESMFold, AlphaFold2, AlphaFold3, and RoseTTAFold, along with the rebuilt model structures and electron density maps, were deposited in Zenodo (https://doi.org/10.5281/zenodo.20276002).

## Supplementary Materials

The following supporting information can be downloaded at: https://www.mdpi.com/article/10.3390/ijms27156553/s1.

## Abbreviations

The following abbreviations are used in this manuscript:

AI	Artificial intelligence
ESM	ESMFold
AF2	AlphaFold 2
AF3	AlphaFold 3
RF	RoseTTAFold
ALAD	δ-aminolevulinic acid dehydratase
pLDDT	predicted Local Distance Difference Test
