# Supplementary material for: AI-Predicted Model-Guided Rebuilding of the Experimental Structure of Mouse δ-Aminolevulinic Acid Dehydratase
Source: Int J Mol Sci. 2026 Jul 23;27(15):6553. doi: 10.3390/ijms27156553 (PMC13465085; doi:10.3390/ijms27156553)
Supplement: Supplementary file 1 [file ijms-27-06553-s001.zip › ijms-4355730-supplementary.pdf]

Supplementary data

AI-Predicted Model-Guided Rebuilding of the Experimental Structure of Mouse δ-Aminolevulinic Acid Dehydratase

Ki Hyun Nam*

College of General Education, Kookmin University, Seoul 02707, Republic of Korea

*Correspondence: structure@kookmin.ac.kr

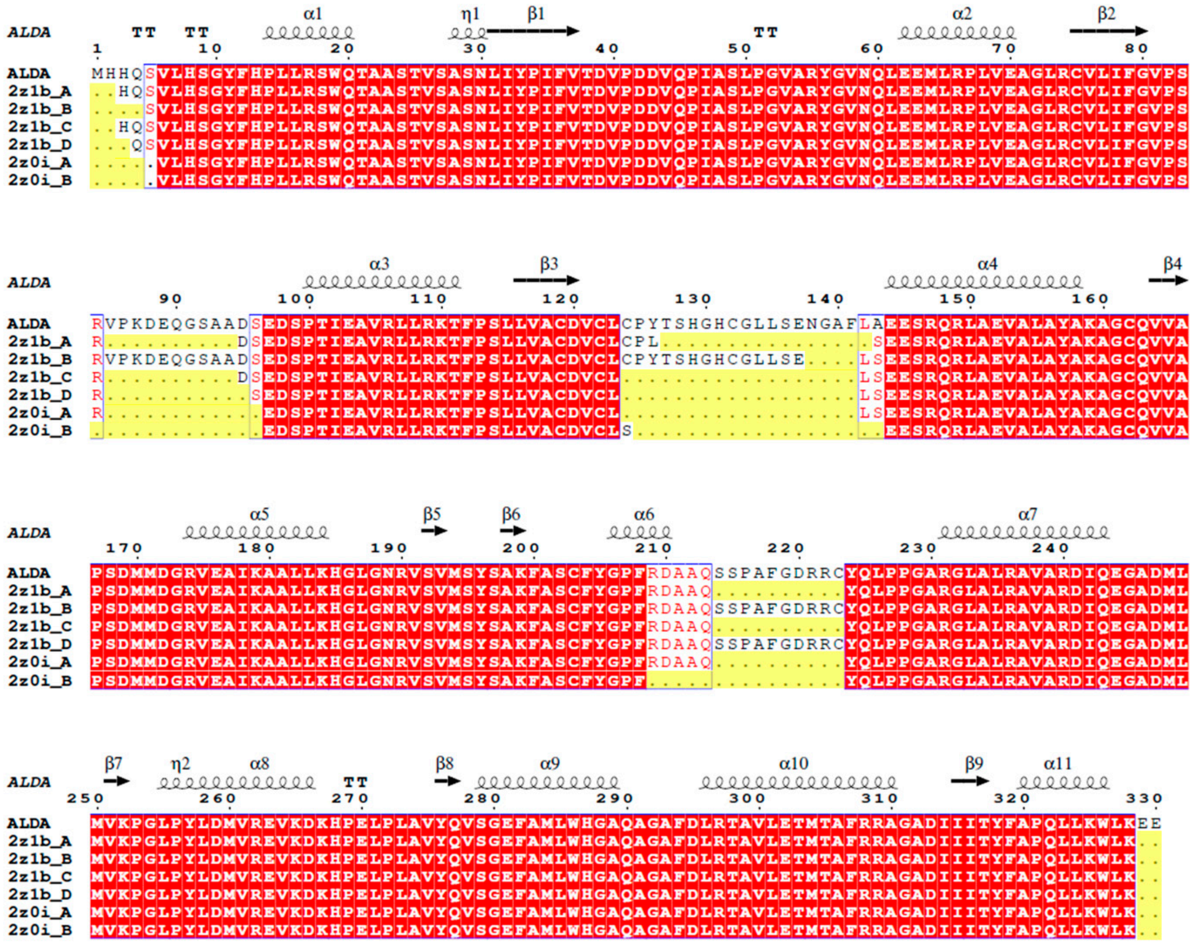

Supplementary Figure S1. Sequence alignment of the ALAD molecules from PDB IDs 2Z1B and 2Z0I. Missing-residue regions in the deposited model structures are highlighted in yellow. Selenomethionine residues in 2Z0I are denoted by M.

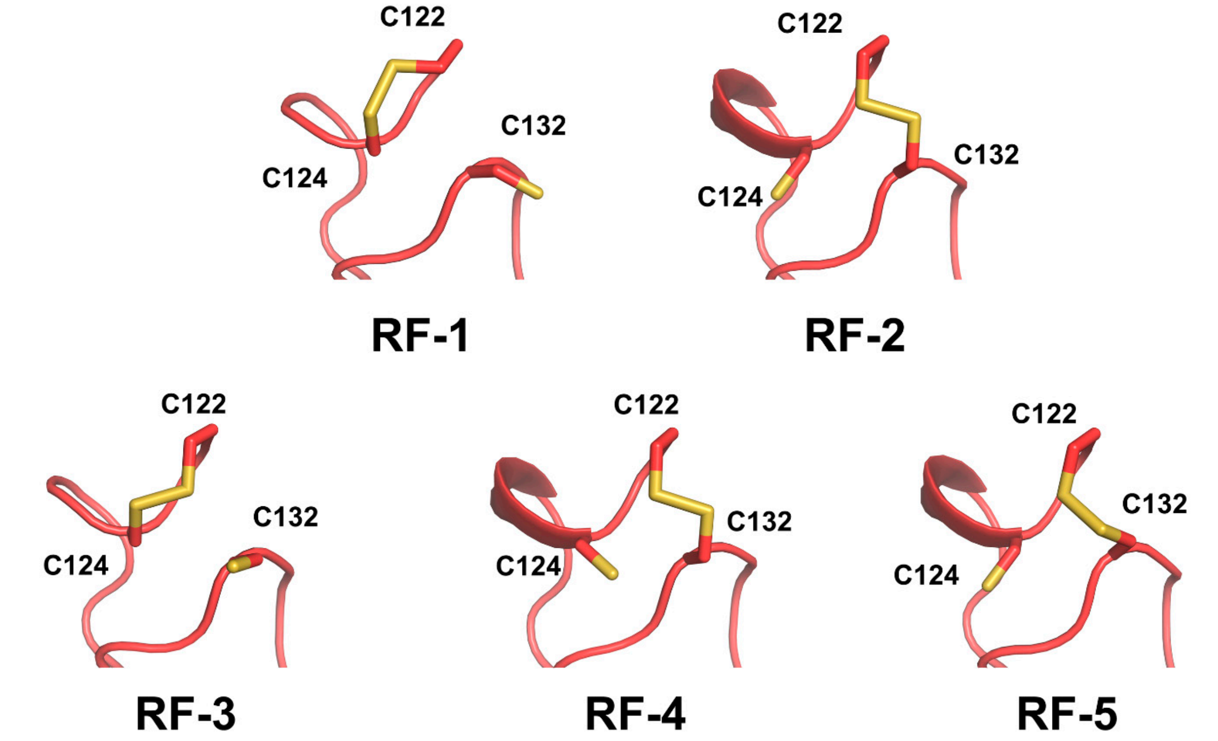

Supplementary Figure S2. Disulfide bond formation at the zinc-binding cysteine residues in the RF-generated ALAD model structures.

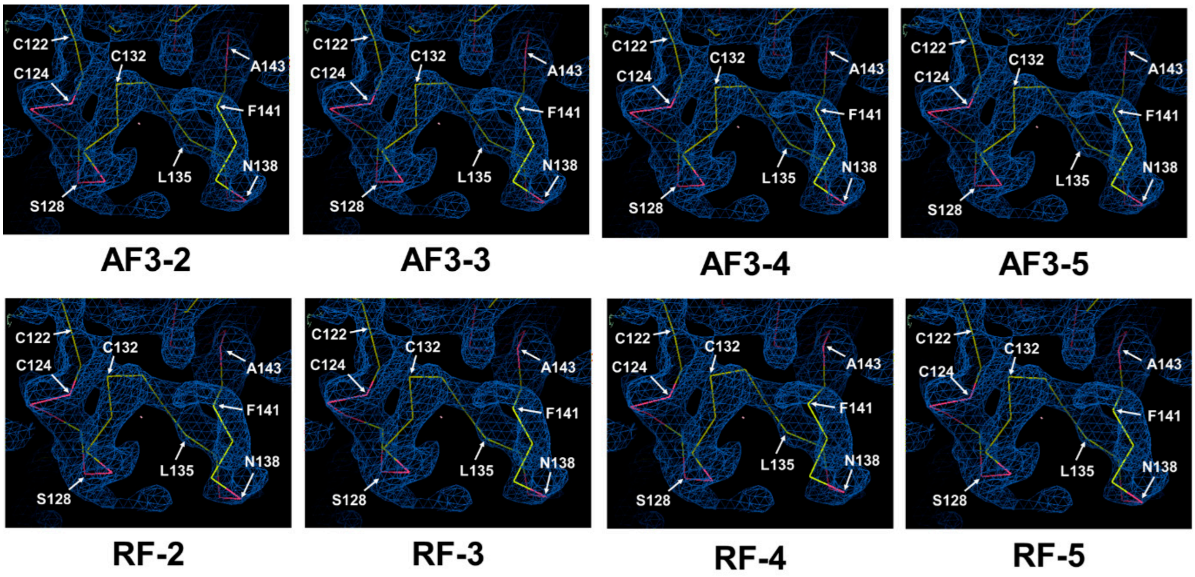

Supplementary Figure S3. Superimposition of the AF3- and RF-predicted ALAD models onto the experimental structure. The overlay shows that the AI-predicted models fit well within the 2Fo−Fc electron density map (blue mesh, contoured at 0.9σ) of the original experimental ALAD structure.

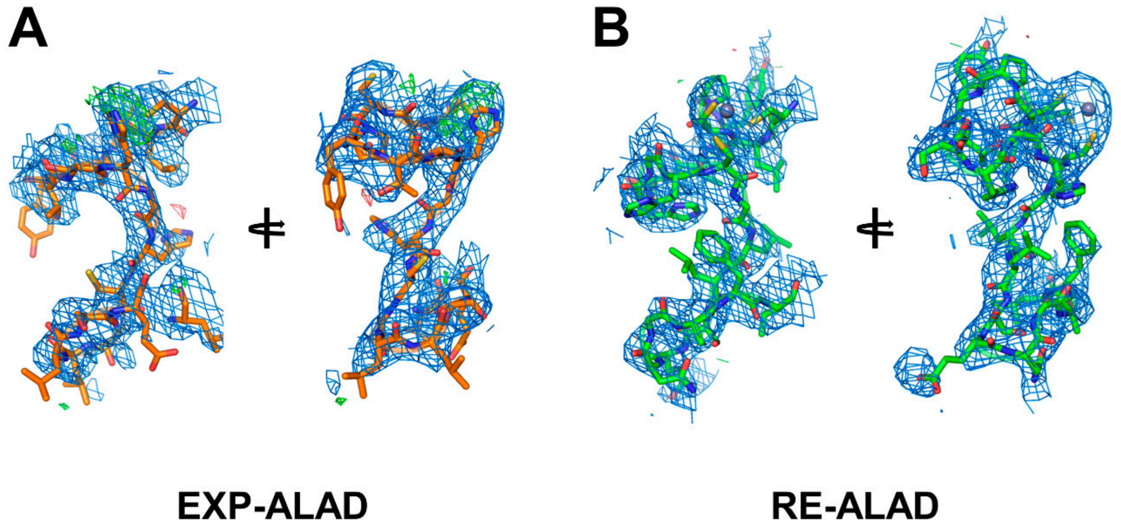

Supplementary Figure S4. 2Fo−Fc (blue mesh, +0.8σ) and Fo−Fc (green mesh, +3σ; red mesh, −3σ) electron density maps for the rebuilt region between Cys122 and Leu142 in (A) EXP-ALAD and (B) RE-ALAD. 2Fo−Fc (blue mesh, 0.87σ) and Fo−Fc (green mesh, +3σ; red mesh, −3σ) electron density maps for all molecules in (C) EXP-ALAD and (D) RE-ALAD.

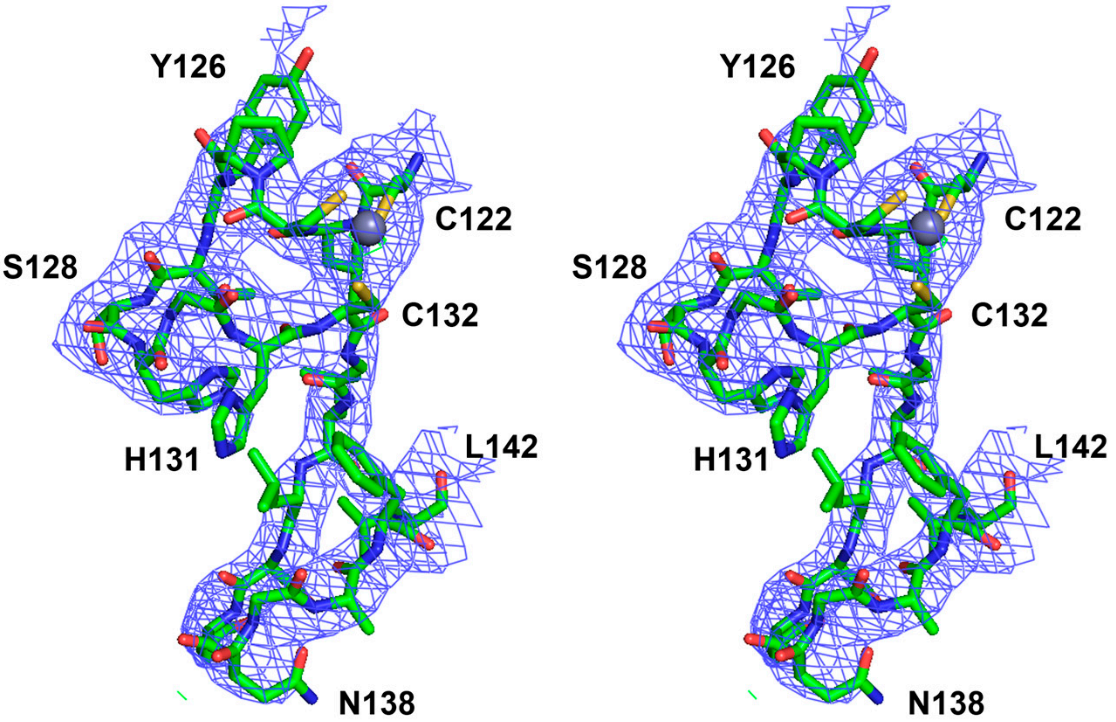

Supplementary Figure S5. Stereo-view of the rebuilt loop (Cys122–Leu142) in RE-ALAD superimposed on the 2Fo−Fc electron density map (blue mesh, +1σ) and the Fo−Fc electron density map (green mesh, +3σ; red mesh, −3σ).
